# Silk-Based Biomaterials for Designing Bioinspired Microarchitecture for Various Biomedical Applications

**DOI:** 10.3390/biomimetics8010055

**Published:** 2023-01-28

**Authors:** Ajay Kumar Sahi, Shravanya Gundu, Pooja Kumari, Tomasz Klepka, Alina Sionkowska

**Affiliations:** 1 Faculty of Chemistry, Nicolaus Copernicus University in Torun, Jurija Gagarina 11, 87-100 Toruń, Poland; 2 Indian Institute of Technology, School of Biomedical Engineering, Banaras Hindu University, Varanasi 221005, Uttar Pradesh, India; 3 Department of Technology and Polymer Processing, Faculty of Mechanical Engineering, Lublin University of Technology, 36, Nadbystrzycka Str, 20-618 Lublin, Poland; 4 Calisia University, Nowy Świat 4, 62-800 Kalisz, Poland

**Keywords:** bioinspiration, biomaterials, silk fibroin, tissue engineering, scaffolds

## Abstract

Biomaterial research has led to revolutionary healthcare advances. Natural biological macromolecules can impact high-performance, multipurpose materials. This has prompted the quest for affordable healthcare solutions, with a focus on renewable biomaterials with a wide variety of applications and ecologically friendly techniques. Imitating their chemical compositions and hierarchical structures, bioinspired based materials have elevated rapidly over the past few decades. Bio-inspired strategies entail extracting fundamental components and reassembling them into programmable biomaterials. This method may improve its processability and modifiability, allowing it to meet the biological application criteria. Silk is a desirable biosourced raw material due to its high mechanical properties, flexibility, bioactive component sequestration, controlled biodegradability, remarkable biocompatibility, and inexpensiveness. Silk regulates temporo-spatial, biochemical and biophysical reactions. Extracellular biophysical factors regulate cellular destiny dynamically. This review examines the bioinspired structural and functional properties of silk material based scaffolds. We explored silk types, chemical composition, architecture, mechanical properties, topography, and 3D geometry to unlock the body’s innate regenerative potential, keeping in mind the novel biophysical properties of silk in film, fiber, and other potential forms, coupled with facile chemical changes, and its ability to match functional requirements for specific tissues.

## 1. Introduction

Following millions of years of evolution, the structures and functions of the lifeforms found in nature have nearly reached their full potential [1,2]. A human body comprises a variety of complex tissues, all of which have hierarchical structures ranging from the macroscopic to the nanoscale in order to keep the body functioning properly [3,4]. In order to fabricate materials with desirable characteristics and properties to withstand demanding applications, it is indispensable to have a clear understanding of the complex functions that are exhibited by the particular material and processes that are found in nature. There are still a lot of obstacles to overcome in the research and applications of biomaterials, even though biomaterials have been used to improve human health to a significant degree and have seen a lot of accomplishment in doing so. For example, the majority of synthetic materials used in medical devices elicit non-specific responses from biological systems upon implantation. These responses further trigger a cascade of undesirable reactions, such as clotting or thrombus formation, inflammatory responses, foreign body reactions, and rejection [5,6,7,8]. Over the course of several decades, significant developments in biomaterial research have led to the development of ground-breaking technologies that have been applied to the field of healthcare [9]. This has sparked a search for low-cost healthcare solutions, with a particular emphasis on environmentally friendly biomaterials that have a wide range of uses and are manufactured using environmentally responsible processes. The process of drawing ideas and inspiration from biological systems and applying them to technical and design challenges is known as bioinspiration. It has two more noteworthy properties besides its ability to spark new thoughts. It can identify easy study topics and places where results can lead to practical function more immediately than in certain popular chemical disciplines. Biological materials serve as inspiration for high-performance, widely-used materials. The use of bioinspired materials has developed rapidly by replicating their chemical compositions and hierarchical structures [10,11]. Due to the numerous instances of complex structures and designs that have developed over billions of years in a variety of situations, nature serves as a great source of inspiration for scientific inquiry. Biomaterials for tissue engineering have lately concentrated on the fabrication of biomimetic materials that can elicit specific cellular responses and drive new tissue production mediated by biomolecular recognition. Numerous biomaterials have progressed toward higher levels of functionalities and performances, which can be utilized in the construction of unique biomedical devices thanks to these advancements. In particular, naturally generated biopolymers provide an extensive variety of opportunities for the construction of acceptable substrates for applications involved in tissue engineering and regeneration. This biological design can originate from natural structures and cell environments, as well as biological anomalies such as shark skin or the adhesive proteins in marine mussels [12,13]. Few well-known examples include the development of a biodegradable bandage that is suitable for use in organ and tissue healing over the course of decades and it represents a significant advancement in the field. The scaly texture of a gecko’s foot was used as a model for the creation of these bandages, which were made by utilizing tiny nanostructures [13]. Black phosphorus has a low cytotoxicity and prevents inflammation and toxicity. In general, the biocompatibility, biodegradability, and biosafety of black phosphorus make it a good candidate for use in biomedicine. This includes the use of pharmaceutical applications, scaffolding, prosthetic coatings, biosensing devices, and medical imaging [14]. Similarly, MIT scientists devised a puffer-fish-inspired hydrogel tablet to examine ulcers and cancers, which lasted longer inside the stomach and gastrointestinal tract [15]. In a similar manner, there are a great deal of biomaterials that provide exceptional instances of bioinspiration. In this article, we examine the structural and functional materials based on silk that were inspired by biological systems. Silk is a feasible option for a raw material that is obtained from biomaterials due to its superior mechanical characteristics, biocompatibility, and flexibility.

## 2. Biomaterials

### 2.1. Definition

Tissue engineering is an interdisciplinary field devoted to repair, replace, or regenerate injured or defective organs in the body [16,17]. The body has natural self-healing characteristics, although the level of restoration varies by tissue and by the degree of trauma, illness severity, or extent of restoration required [17,18]. In addition, the proliferation and growth of different cells vary depending on cell specificity [19]. Tissue engineering (TE) encompasses engineering and biological concepts and develops functional tissues by integrating cells, scaffolds, biomaterials, and bioactive compounds. The appropriate contribution of each element relies on the application, approach, and patient demographics such as age, co-morbidities, and other variables [20]. There are two approaches that might be employed when attempting tissue engineering i.e., ex vivo and in vivo. The ex vivo approach encompasses extraction of healthy cellular components from the donor, followed by their culture on an external scaffold in bioreactors to promote cellular proliferation and differentiation. The in-vivo approach is a quick and convenient solution in tissue engineering that avoids cells seeding and includes implanting a prefabricated scaffold, comprising biocompatible biomaterials with a specified dimension equivalent to the injured area, directly into the target tissue. It relies on migration and adhesion of the surrounding cells by stimulating the host tissue regeneration [17]. In-vivo tissue engineering involves biophysical and pharmacological signals to guide endogenous cells to the damage site. These signals drive regeneration by changing the extracellular microenvironment [21]. The prerequisites for biomaterial scaffolds include bio-compatibility and non-toxicity, as well as the biomaterial scaffold’s ability to duplicate the extracellular matrix (ECM), function as a carrier for growth factors, and construct an adequate scaffold for tissue regeneration [17,18]. In order to stimulate tissue regeneration, it is mandatory to design and fabricate an optimal biomaterial that efficiently transports cell populations and therapeutic substances, as well as provides structural scaffolding with adequate mechanical qualities for tissues. The biomaterial degradation rate should be equivalent to that of localized tissue regeneration following implantation [16,18]. Any substance, structure, or surface that interacts with biological systems is considered as a biomaterial. It may be produced from natural or synthetic sources and could be utilized for partial or complete tissue replacement. It is important for an optimal biomaterial to be able to imitate the body, not just in terms of structure but in terms of function [22]. Scaffold materials must fulfil various tissue engineering requirements, such as the substrate materials must be biocompatible. The material must not cause any inflammatory reaction, immunogenicity, or cytotoxicity upon implantation. Tissue scaffolds must be easily sterilizable to avoid infection [23]. The scaffold’s mechanical characteristics must be sufficient to prevent structural failure during handling and patient activity [24]. In addition, biomaterials must be able to exhibit a temporary three-dimensional (3D) support in order to interact biomolecularly with cells in order to influence their function, hence directing the spatially and temporally complicated multicellular processes of tissue development and regeneration [25].

### 2.2. Biobased Biomaterials

**Figure 1:**
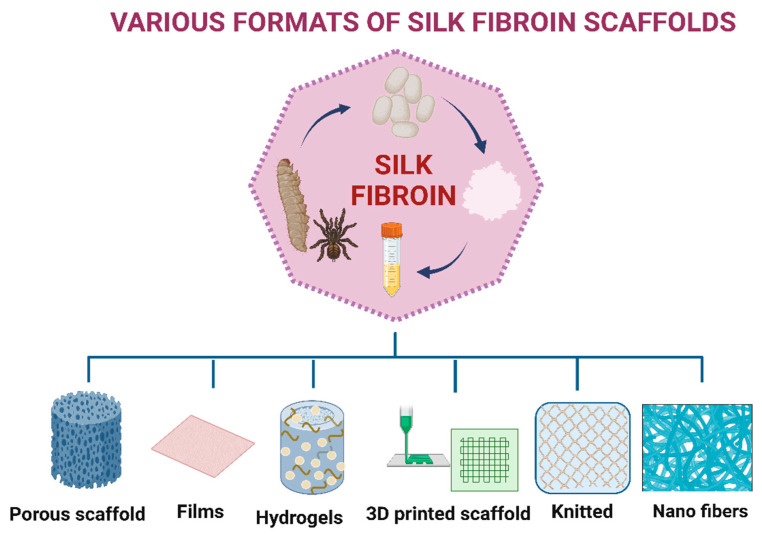
Silk biomaterial-based various 3-dimensional (3D) scaffolds/products that may be have tissue engineering applications.

Biomaterials may be either natural or synthetic in origin. Both naturally produced and synthetic materials have been the subject of extensive study and attention. Despite the benefits of synthetic materials, such as greater control over material features and tissue interactions, developing synthetic biomaterials as cellular substrates exhibit certain limitations, i.e., purification, immunogenicity, and pathogen transmission [26]. Natural polymers, such as chitosan, gelatin, collagen, silk, cellulose, and alginates, are preferred over synthetic polymers, because natural polymers exhibit better biocompatibility, superior biodegradability, and lower toxicity compared to synthetic materials [17,27,28,29,30]. Biologically derived materials can improve cell adhesion and migration, inducing extracellular matrix production and promoting tissue healing [31]. Collagen is the most prevalent protein class in the human body and possesses numerous favorable characteristics, including biodegradability, biocompatibility, simple accessibility, and adaptability. Nevertheless, because collagen is a protein, it remains difficult to sterilize without any alteration in the architecture and it is difficult to maintain its stability [32]. Similarly, gelatin is also a collagen derived natural biopolymer featuring biocompatibility, water solubility, low immunogenicity, flexibility, adhesiveness, cell adhesion, growth, and cost-effectiveness, as well as the capacity to make transparent architecture. However, gelatin has weak mechanical capabilities and degrades into a colloidal solution at temperatures above physiological body temperature, i.e., 37 °C [29,33]. Chitosan is a naturally occurring polycationic linear polysaccharide that results from the partial deacetylation of chitin. Few studies have described chitosan as a potential 3D scaffold regeneration material. The porous structures of chitosan facilitate cell growth, motility, and nutrition exchange [34]. Moreover, the controlled porosity of chitosan scaffolds promotes angiogenesis, which is essential for soft tissue regeneration [34,35,36]. There are several limitations of chitosan; for example, chitosan lacks cell adhesion moieties and the variable composition of glucosamine and acetyl-glucosamine and the molecular weight distribution adds difficulty to the process of identifying favorable qualities in the finished product [36]. Similarly, several natural biomaterials have demonstrated positive results for the development of scaffolds, but they also have a number of limitations. These limitations can be circumvented by blending or mixing them in certain composition that enables the material’s effectiveness for tissue regeneration. Silk has demonstrated a great potential to be used as a biomaterial for tissue regeneration among the excellent naturally available biomaterials. Silks are well-known natural fibers that are generated by a number of silkworm insects and spiders, including *Bombyx mori*, which is one of the sources that has received the most attention from researchers (Figure 1) [9,37,38]. Despite the fact that it has a number of benefits, silk also has a number of drawbacks. Signaling, extracellular matrix deposition, cell proliferation, and differentiation can occur when cells adhere to surfaces and release active compounds. The process of cell attachment is complicated since it is influenced by several factors, including cell behavior, the properties of the surface, and environmental conditions. Furthermore, the surface properties of a biomaterial comprise charge, chemical composition, hydrophobicity, softness, and roughness [39]. Pure silk lacks bioactive components to interact with the cell receptors for the activation of the tissue repair process [40]. As a result of the limited amount of carboxyl groups in the side chains of amino acids in silk fibroin, it cannot be used for chemical modification [41].

## 3. Silk

### 3.1. Global Potential of Silk as a Biopolymer

China is the greatest producer of silk in the world, accounting for ~79–80% of the total raw silk output globally. India accounts for ~13–15% of the overall production of raw silk across the world, making it the second largest producer after China. Together, these two countries are responsible for almost 93–95% of the total silk output globally [42,43,44]. Mulberry silk, which is generated by the silkworm *B. mori*, is the type of silk that is produced and used in greater quantities than any other variety [45].

### 3.2. Silk Characteristics

**Figure 2:**
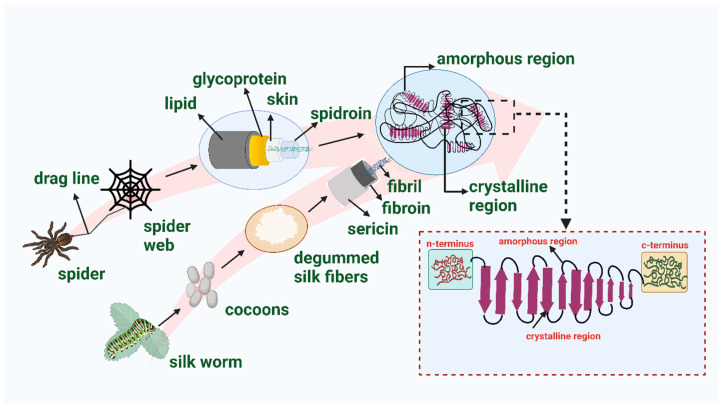
The internal architecture of silk fibroin by dissolving sericin protein exposing silk fibroin.

Silk, a naturally occurring structural protein, is an example of special category of biocompatible and environmentally friendly polymers. As a result of its biodegradability, minimal immunogenic reaction, and ease of processing, it has been the subject of considerable attention in the field of biomedical research [9]. Spiders and silk worms are two primary sources of silk. The majority of arthropods spin silks that are then utilized to construct elaborate traps and nests to safeguard their young from predators and harmful elements [46]. Spider silk, in particular, is generated by massive ampullate glands, and the cocoon silk of *Bombyx mori* has attracted the most attention and research [46,47,48]. Spider silk is one of the most amazing, hierarchically organized naturally occurring, environment friendly material, and has remarkable material qualities including high toughness, high stiffness, exceptional extensibility (~30% elongation to fracture), and biocompatibility [48,49,50]. In addition, high strength-to-density of silk dragline ratios indicate high energy impact absorption. Silk fibroin, which comes from a variety of silkworm and spider species, has been the subject of research for their potential applications in the medical field. Some of these applications include tissue-engineered grafts, cancer therapeutics, high-throughput tissue-on-chip models, food preservatives, biomedical imaging, biosensing, biomedical textiles, implants, cosmetics, and bioremediation. Because of these characteristics, spider silk is one of the most viable materials for tissue regeneration, where mechanical stability is a crucial prerequisite [48]. However, wide-scale manufacturing of silk requires enormous quantities of silk, since spiders can only produce little amounts of silk, which cannot be spun into a single homogenous fiber (since spiders employ several types of silk). In addition, spiders are predatory and cannibalistic, which impedes the formation and maintenance of dense populations. In addition, the fibers of natural dragline silk are heterogeneous. Variability in fiber design enhances fiber strength; however, high heterogeneity may result in less resilient fibers [48,51]. Thus, using silkworms, and particularly mulberry silkworms, for mass manufacturing of silk is a viable option. The economic significance of the mulberry silkworm (*Bombyx mori*) lies in the fact that it may be reared in captivity [52,53]. There is the high possibility of using silk protein as a potential biomaterial for implantation due to its favorable properties. These include a high tensile strength, ease of handling, quick and inexpensive processing, high availability, controllable dimensional and material characteristics, the ability to promote cell growth, being sterilizable, and transparency, as well as the significant number of studies already showing its acceptability in-vivo [54,55,56]. Silkworms, which are the primary source of silk, are typically classified into two categories based on their feeding habits i.e., those feeding on mulberry leaves are known as mulberry silkworms (e.g., *Bombyx mori*) and those that do not feed on mulberry leaves are termed as non-mulberry silkworms (e.g., *Antheraea mylitta*, *Antheraea assama*, and *Samia ricini*). Mulberry silkworms, also known as *B. mori* (which are members of the family Bombyciidae), are responsible for approximately 90% of the world’s total output of silk [57]. Silk fibroin (SF) and silk sericin protein are the two constituents that make up the protein biopolymer that is obtained from silkworm cocoons. While SF is responsible for providing silk with its tensile strength and load-bearing capacity, sericin is responsible for the adhesive properties. Sericin is a form of glycoprotein responsible for 25–30% of the overall weight of cocoons. Silk fibroin is a protein that is fibrous and has a semi-crystalline structure (Figure 2). Silk fibroin is composed of a light (L) chain (~25 kDa) and a heavy (H) chain (~390 kDa) polypeptide that are bonded together by a single disulfide bond at the C-terminus of the H-chain to generate a H–L complex. Silk fibroin is responsible for silk’s characteristic rigidity and strength; more specifically, the H chain (primarily comprising the three simplest amino acids, i.e., glycine (~43–46%), alanine (~25–30%), and serine (S) (~12%) of the polypeptide is responsible for the high mechanical strength [47,58,59]. The amino acid sequence of the L-chain comprises non-repeating subunits; therefore, the L-chain has a higher degree of hydrophilicity and is elastic in nature. In raw silk, the protein sericin is arranged so that it covers the surface of two fibroin fibers running in parallel and binds them together. Sericin is an amorphous protein that has the consistency of glue and works as an adhesive binder to ensure that the fibers maintain their structural integrity. Because sericin is water-soluble, it may be eliminated using a degumming process [58,59].

### 3.3. Extraction of Silk Fibroin

**Figure 3:**
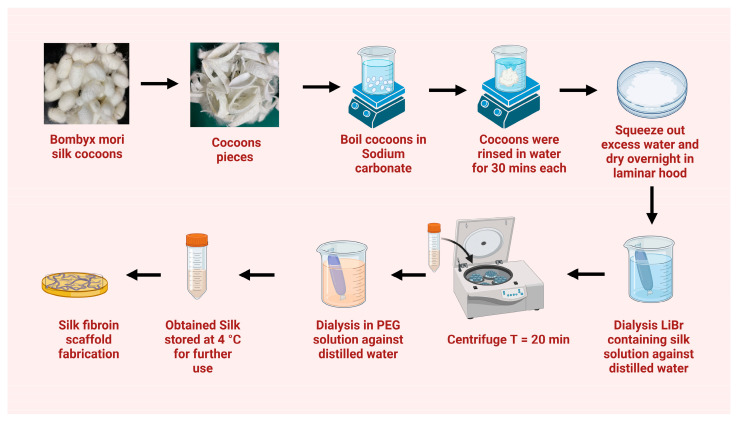
Schematic representing the extraction of silkworm silk (*B. mori*), using the typical sodium carbonate/lithium bromide (LiBr) method.

It is necessary to first separate the two protein components that make up the silk thread in order to create a pure silk fibroin solution. The separation of fibroin by solubilizing the sericin in water is termed as degumming. Degumming on a laboratory scale can be accomplished using a wide variety of techniques, including the application of highly concentrated urea, proteases, bromelain, biosurfactants, and organic acids such as boric acid sodium borate buffer, or citric acid, as well as infrared heating and microwave irradiation [60,61,62,63]. The most accepted technique for degumming involves boiling the silk thread in a buffer containing 0.02 M sodium carbonate (Na_2_CO_3_) for 0.5 to 1 h, followed by repetitive washing to remove sericin completely. The boiling duration and salt concentration must be precisely determined, since extended boiling or excessive salt concentrations cause a change in molecular weight owing to the fragmentation of silk fibroin, which in turn decreases the physical qualities of the degummed silk fibers [64,65,66]. The hydrogen bonding and β-sheet crystallites of silk fibroin prevent it from dissolving during the degumming process. Therefore, aqueous or organic salt-containing systems with high ionic strength are most suitable for the complete dissolution of silk fibroin. A few examples are lithium bromide solution with water, N-methyl morpholine (NMMO), calcium chloride-formic acid, lithium thiocyanate, and calcium chloride-ethanol-water-based ternary solution system. The most frequent method for dissolving degummed silk fibroin fibers is a lithium bromide-water-based method. Briefly, degummed silk fibers are air-dried overnight and then dissolved with LiBr at 60 °C for four hours with periodic shaking. The highly viscous silk solution is dialyzed against ultrapure water until all the salt has been removed in order to regenerate the silk. The regenerated silk is further stored at 4 °C for direct usage as a solution or can be lyophilized to store for longer duration in the powder form (Figure 3) [60,61,62,65]. Moreover, regenerated silk solutions are becoming increasingly popular as a means of fabricating a variety of bioinspired biomaterials in the form of gels, films, membranes, nanofibers, solid freeform fabricated scaffolds, and sponges.

## 4. Development of 3D Microarchitecture Biomaterials

**Figure 4:**
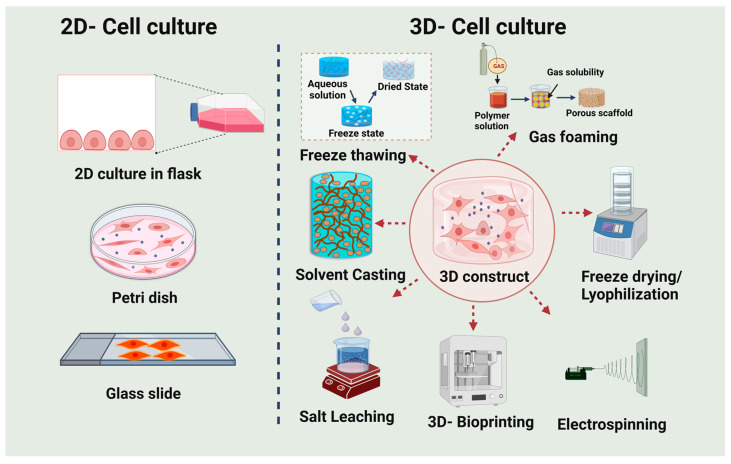
The 2-dimensional (2D) and 3-dimensional (3D) cell culture system and the various techniques used to fabricate 3D materials/scaffolds for tissue engineering applications.

Conventional two-dimensional cultures are used for most cell-based experiments. In these cultures, cells are grown as a monolayer on flat and rigid surfaces by adhering to a flask or petri dish. Despite the fact that 2D cell culture has proven to be an effective technique for cell-based research due to the convenience of maintaining cell cultures, its limitations are becoming more apparent as the cells do not imitate native tissue and have limited access to oxygen and growth factors of cultured media [67]. Sometimes, 2D cell culture assays can indeed be inaccurate and are not attributable for in vivo responses. Many clinical attempts continue to fail due to the data acquired from the 2D culture assay, i.e., microenvironment, in adequate space for cells to grow and contaminate. Cell-based systems stimulate the activity of cells in vivo and deliver more predictable in vivo assay outcomes. Recent research suggests that 3D cell culture techniques reflect the tissue microenvironment more precisely than 2D methods, since cells in the in vivo environment are surrounded by other cells and extracellular matrix (ECM) in a three-dimensional (3D) manner and 3D-cultured cells behave more similarly to in vivo cells (Figure 4). The 3-D model incorporates cell-to-cell and cell-to-ECM interactions, tissue-specific toughness, nourishment, oxygen, and wastage elimination [67,68]. In fact, 3D-cultured cells vary morphologically and physiologically from 2D-cultured cells, which does not profoundly account for the natural 3D environment of the cells [67,69].

## 5. Various Approaches for Fabricating 3D Microarchitecture Scaffolds

### 5.1. Hydrogels

Hydrogels have emerged as one of the most famous and diverse groups of materials utilized in tissue engineering [70]. Hydrogels are three-dimensional networks of cross-linked, hydrophilic polymers that can retain a significant quantity of water in a swelled scaffold [71]. The formation of hydrogels results in extremely porous networks, which allow for the diffusion of nutrients, oxygen, and biomolecules, while also permitting for the transfer of byproducts and toxins away from cells. The porosity of the material is often sufficient to allow for cell penetration and interconnectivity, resulting in a good growing medium for tissue [72]. In a study, Yao et al. created an all-natural green hydrogel integrating biliverdin with silk fibroin for anti-glioma photothermal therapy (PTT) and tissue regeneration. With biliverdin, bioinspired green hydrogel (BVSF) could fast reach 45 °C under NIR irradiation, killing cancer cells in vitro and inhibiting tumor development in vivo. thereby producing hydroxyapatite deposition in collagen gels and permitting MSC adhesion, proliferation, and osteogenic differentiation in vitro [11]. Griffanti et al. developed an injectable dense collagen hydrogel containing silk extracted incorporation (SS). Incorporating SS into collagen gels enhanced the deposition of hydroxyapatite and promoted the adhesion, proliferation, and osteogenic differentiation of MSCs in vitro, which have shown that collagen-containing SS have the potential as scaffolds for bone tissue engineering applications [73]. The hydrogels’ unique properties and applications have garnered attention in recent years. Hydrogels are biocompatible and non-toxic because their polymers imitate the extracellular matrix of living tissues. They are suitable for wound healing and tissue engineering [74]. Hydrogels are elastic and mechanically comparable to real tissues due to their high-water content. This reduces tissue damage and improves hydrogel-based device performance [75]. Hydrogels can release medicines or other bioactive molecules slowly, improving therapy efficacy and reducing negative effects [76]. Despite these advantages, hydrogels have limitations such as being weaker and less rigid than conventional polymers, which makes them unsuitable for applications requiring great strength or durability [77]. Frequently, hydrogels are created using complicated processes, making their production laborious and expensive. This can restrict their applications and commercial uses [78]. Hydrogels are susceptible to variations in temperature and pH, which can result in instability and deterioration over time [79]. This can limit their long-term performance and usability.

### 5.2. Electrospinning

Electrospinning is a manufacturing technique that uses an electrostatically driven process to produce electrospun fibers, which resembles nanofibrous yarn. The thickness of these fibers is generally found in the range of a few nanometers to a few micrometers [80]. When a relatively high voltage is applied to a droplet of liquid, the liquid’s body becomes electrically charged. This causes electrostatic repulsion to subdue over surface tension, which causes the droplet to expand and form a Taylor cone, which is an important part of the process of liquid stream ejection. The trajectory of the jet initially follows a straight line, but it quickly begins to zigzag erratically due to bending instabilities. When the jet is shrunk down to tiny dimensions, the fluid quickly solidifies, leaving behind a deposit of solid nanofibers on the collector that is grounded. Because of its versatility and ease of manufacture of continuous nanofibers from a wide range of materials, electrospinning is often regarded as the most successful way of producing nanofibers [81]. The choice of collector influences the fiber orientation and the structure of a construct. Additionally, the diameter and architecture of the fibers are dependent on several parameters, including the distance between the collector and needle tip, the rate of polymer ejection, applied voltage, nozzle diameter of needle, type of solvent used, and the polymer concentration [82,83]. Due to the outstanding properties that electrospun materials possess, such as high porosity, small diameter, good pore interconnectivity, and a high surface-to-volume ratio, these materials have acquired significant interest in a wide range of industries [84,85]. Electrospun products have a variety of applications in the field of food packaging, preservatives [85], sensors, filters, solar cells, fuel cells, batteries [86], drug delivery, variety of tissue engineering applications, reinforced materials [87], and energy generation [88]. Electrospun silk along with gelatin have been utilized for several 3D scaffolds for tissue engineering applications such as wound dressing material, cornea stroma equivalent, and tendon regeneration [29,82,85,89,90,91,92]. Electrospinning has several drawbacks, including poor cell penetration and migration due to scaffold fiber packing, residual solvent toxicity, and inadequate scaffold mechanical strength for load-bearing applications. All of these drawbacks are caused by the fact that electrospinning is performed on a very small scale [93].

### 5.3. Salt Leaching

Another process that produces highly porous foams is solvent casting/particulate leaching. A porogen is introduced to the polymer solution and leached out once the polymer has dried. A porogen is a substance that dissolves in the polymer’s non-solvent. The porogen is leached simply by dissolving it in its solvent (the most commonly used solvent is water). As a result, the residual polymer structure has a large number of linked holes that the porogen left behind. This technique is chosen because the pore diameters may be adjusted by the porogen used as well as its size and quantity [94,95]. In spite of the fact that salt-leached silk scaffolds have been employed in tissue engineering, their uses in the regeneration of softer tissues may be hampered by their extreme rigidity. Silk-bound water interactions could be managed by regulating processing in order to enable the fabrication of salt-leached porous scaffolds with adjustable mechanical characteristics [96]. Silk fibroin with varied nanostructures were self-assembled in aqueous solution as a consequence of repeated drying and dissolving processes. A theory for regulating the porosity architectures of freeze-dried silk fibroin scaffolds has been revealed, suggesting that the nanostructures of silk fibroin in solution play a crucial role in porous scaffold development [97]. This process has a number of benefits, such as being easy to perform and requiring little equipment. In addition to this, it creates scaffolds that are very porous and provide the ability to change and regulate the size of the pores. Still, the only shapes that can be made with scaffolding are sheets and tubes, which is a limiting factor [98]. When using particle leaching, it is impossible to build a well-interconnected porous structure that has a regular and reproducible structure. This is one of the most significant drawbacks of using salt leaching [99].

### 5.4. 3D Bioprinting

The advancement of additive manufacturing methods collectively referred to as 3D printing has enabled the production of scaffolds with extremely thin structures and complicated geometries by utilizing computer-aided design (CAD) data obtained from patient medical imaging. Solid freeform technology is transforming technology and holds tremendous promise for fabricating highly ordered biodegradable scaffolds for damaged tissues and organs [100]. Stereolithography (SL), fused deposition modeling (FDM), selective laser sintering (SLS), and 3D printing are all solid freeform fabrication (SFF) techniques that can be used to develop scaffolds for various tissue engineering applications [101,102]. In general, 3D bioprinting uses a layer-by-layer approach to deposit materials called bioinks to build tissue-like structures that may be utilized in a variety of medical and tissue engineering applications [103]. Three-dimensional bioprinting encompasses a diverse variety of bioprinting methods and biomaterials, and by using these approaches, researchers may manipulate the characteristics of the scaffold such as the pore size, porosity, and interconnectivity. In comparison to non-biological printing, 3D bioprinting entails extra complexity, including material selection, cell type selection, growth and differentiation factors, and technological problems associated with the sensitivities of live cells and tissue building. Three-dimensional bioprinting has been utilized to generate and transplant a variety of tissues, including multilayered skin, bone, vascular grafts, tracheal splints, cardiac tissue, and cartilaginous structures [104]. Bioprinting applications fall into two broad categories: (1) tissue regeneration, which includes printing blood vessels, heart valves, musculoskeletal tissues, liver, nerves, and skin; and (2) biomedical applications, which include drug development and screening [105]. One of the major advantages of 3D bioprinting is the fabrication of highly specialized and precise tissue architectures. These structures may be placed to use in a number of contexts, such as in the testing of drugs, the engineering of tissue, and the practice of regenerative medicine. The process of tissue engineering may be greatly sped up by 3D bioprinting, which in turn makes it possible to produce functional tissue structures more quickly. Traditional techniques of tissue engineering, such as using animal models or human cadavers, are associated with a higher cost and greater ethical problems than 3D bioprinting, which has the potential to reduce each of these aspects. The ability to fabricate tubular structures with multi-layer free-form constructs is the most significant advantage that 3D bioprinting technology brings to tubular tissue engineering. Other advantages include the ability to place biomaterials within a cell and printable ink that contains biochemical microenvironmental cues [106,107]. However, there are several downsides, such as the necessity of using a substance with a low viscosity in order to prevent clogging, and the requirement that bioprinting material should be swiftly gelated in post-print in order to form 3D models [108]. The process of bioprinting intricate and functioning tissue structures is difficult, since it calls for a high degree of knowledge as well as specific pieces of equipment. Additionally, the variety of the cells and biomaterials that are employed in the bioprinting process might have an effect on the final tissue’s quality as well as its functioning. The current state of 3D bioprinting has restrictions in terms of the scale and resolution, both of which can have an impact on the printed tissue structures’ ability to function and their level of development. The advent of 3D bioprinting presents a number of regulatory and ethical difficulties, including the requirement for appropriate governance and rules for the utilization of printed tissue structures in clinical settings [109,110,111].

### 5.5. Gas Foaming

Gas foaming is a solvent-free method of synthesizing bio-artificial matrices. As a result, it is an effective approach for integrating sensitive compounds into matrices while maintaining their bioactivity [112]. The technique of gas foaming has been created in order to eliminate the need for high temperatures and organic solvents that are cytotoxic. This technique involves the use of relatively innocuous gas foaming agents, such as carbon dioxide or nitrogen, in order to pressurize a modelled biodegradable polymer with fluoroform or water until it is saturated or filled with gas bubbles. [113]. For gas foaming, the polymers are molded into a solid disc and then penetrated with carbon dioxide under high pressure for several days in a confined chamber. The gas filters in the polymer during this phase and develops pores for tissue ingrowth [114]. For example, Giannitelli et al. effectively constructed polyurethane-based scaffolds with a thick shell and a porous core for bone regeneration applications using gas foaming, allowing for tissue ingrowth and bone regeneration [115]. The disadvantage of this approach is that the product created may have a closed pore structure or a solid polymeric structure because of inadequate treatment in some cases, which is undesirable. This method usually results in structures that resemble sponges, with pores ranging in size from 30 to 700 microns in diameter and porosity of up to 85–93% [116]. Gas foaming produces high (up to 93%) porosity and may modify pore sizes by changing temperatures and pressures parameters [117]. Pore creation does not happen in the same way every time the procedure is employed. This technology has been linked to a leaching process in order to build better interconnected pores in the gas-foaming procedure [118]. Depending on the rheological qualities of the liquid polymer that is being foamed, this method may have limitations in the management of the pore size, pore interconnectivity, and spatial homogeneity of the material. These limitations might be a problem [119,120].

## 6. Bioinspiration of Silk for Biomedical and Tissue Engineering Applications

### 6.1. Bioinspiration of Bone Tissue Engineering

**Figure 5:**
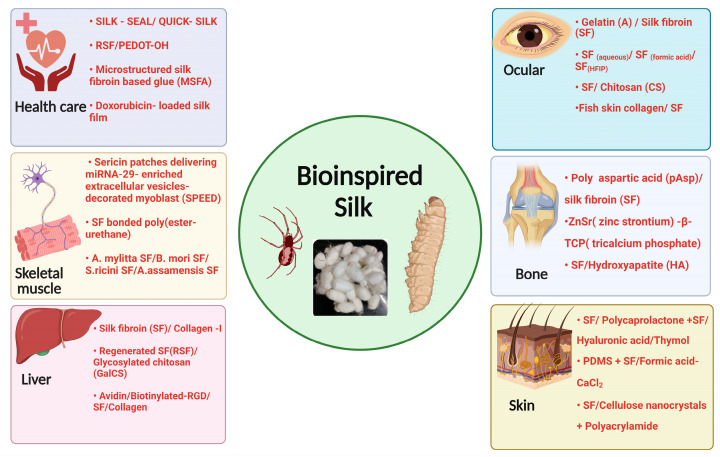
Bioinspired silk-based scaffold/materials utilized for tissue engineering applications of various organs.

Bone tissue engineering is a rapidly evolving field to repair, replace or regenerate injured bone tissue. Natural bones are mineralized connective tissue that is complex and extremely dynamic, consisting of the skeleton and bone marrow. These tissues are well-organized with multiple mechanical and metabolic activities that are sustained by the process of bone remodeling. The extracellular matrix (ECM) comprises 40% organic phase, which includes type I collagen fibrils, growth factors, and proteins, and 60% inorganic phase with hydroxyapatite crystals and non-collagenous proteins that are organized at multiple hierarchical levels [121]. Although the bone has the ability of self-growing, self-regenerating, and self-healing, large bone abnormalities caused by various conditions, such as unintended trauma, congenital disorders, cancer surgical removal, and illness, can have detrimental effects on the body’s normal structures and functioning. To effectively treat such extensive bone defects, appropriate materials and approaches for bone regeneration are yet to be discovered and explored. Numerous inspired bone-based composite scaffolds have been fabricated to address bone and tissue applications by modifying the required prerequisites to mimic the native ECM by encompassing physicochemical properties, morphology, material composition, biocompatibility, degradability, and diverse features of the bone [122]. The addition of hydrophilic constituents, such as peptides or amino acids, and growth factors to the surface of the scaffolds enhances cell adhesion on the scaffolds and facilitates cell growth, probably due to electrostatic interaction between the positive charge on the surface and the negative charge on the cell membrane [123,124,125,126]. In addition, Song et al. have fabricated bioinspired scaffolds by blending collagen derived from duck eel (DC) and polyglycolide acid (PLGA) to determine the effectiveness of the scaffold in promoting the differentiation of bone marrow mesenchymal stem cells (rBMSCs) into osteoblasts [125]. Silk is an interesting material due to its remarkable advantages including mechanical properties, fibrous protein structure, type-I collagen, biomineralization ability, and excellent biocompatibility [56,121,127]. Few research studies have been performed on silk fibroin (SF), a bioinspired material that has gained significant attention for biomimetic bone applications. Since silk/HAP composites perform well in bone-tissue engineering, Collins et al., demonstrated the first load-bearing biomimetic bone-like silk/HAP composite scaffold with mechanical characteristics similar to the cancellous bone [128]. As an example, Wildt et al. recently constructed a bioinspired scaffold made of poly-aspartic acid (pAsp) and SF that was mineralized using stimulated body fluid (SBF). Since pAsp is capable of replicating the activity of non-collagenous proteins, it may stimulate the mineralization of intrafibrillar collagen and also improve SF mineral dispersion [129]. In order to determine the load-bearing capability of the material as nonpyrogenic osteoregenerative scaffolds and also to encourage the proliferation of human bone marrow stromal cells (HBMSCs), a silk/calcium phosphate scaffold was prepared by freezing with phosphate, followed by ethanol, and crosslinking with hexamethylene diisocynate [128]. Ribeiro et al. have fabricated bioinspired composite tubular grafts by employing SF and horseradish peroxidase (HRP) holding ZnSr-doped β-tricalcium phosphate (ZnSr-β-TCP) particles towards repairing the anterior cruciate ligament (ACL). SF has the requisite tensile potentials of the ACL, as well as appropriate biocompatibility and slow degradation for the development of tissue ingrowth in vivo [130]. Fitzpatrick et al. used 3D printed SF/HA-based scaffolds for functionalized bone regeneration. The results revealed that the combination of SF and HA exhibited excellent mechanical properties and also meets the requirement of native bone tissue while showing promising results in promoting osteoinduction (BMP2) innervation (NGF) and vascularization (VEGF) [131]. Farokhi et al. reported that SF/HAP scaffolds that were incorporated with SF microspheres and loaded with small amounts of BMP-2 and VEGF produced a synergistic impact on the formation of bones [132]. Currently, many researchers are looking at the possibility of employing spider silk through the use of 3D bioprinting as a potential route to properly mimic the fine structures of bones in the future (Figure 5).

### 6.2. Bioinspiration of Liver Tissue Engineering

The liver, in contrast to most organs, possesses a remarkable potential to renew itself. Therefore, supportive therapy and extracorporeal devices serve to create an environment that promotes patient self-recovery, which frequently opens the possibility of receiving a liver transplant. While hepatocyte transplantation can be considered an alternative to the transplantation of an entire liver, the lack of adequate numbers of hepatocytes that are able to operate normally and contribute to the therapeutic efficacy prevents its practical deployment. Silk has been recognized as a dominant material over other biomaterials in a variety of different scientific instances. As an example, for the objective of improving mass transfer within 3D scaffolds, a biomimetic vascular tree architecture was fabricated using the same biological characteristics (such as the diameter ratio and branching angle) as a natural vascular system. Rolling a monolayer microstructured scaffold into a 3D silk fibroin-gelatin scaffold was accomplished (Figure 5). Experimental results revealed improved fluid flow via the biomimetic vascular tree network compared to a porous scaffold without channels or branches [133]. Yang et al. studied the cytotoxicity of manufactured SF/gelatin composite scaffolds for liver tissue engineering. Although silk has a number of favorable features, including as strong histocompatibility, biocompatibility, and high mechanical properties, it exhibits hydrophobic characteristics as silk consists of more hydrogen bonds. As a result, pure SF cannot be employed for cell adhesion due to slow degradation. Similarly, gelatin can bond to polymer materials and boost cell proliferation, adhesion, and bioactive components. As a result, the combination of SF and gelatin has shown exceptional levels of biocompatibility for liver tissue engineering [134]. Silk fibroin (SF): gelatin scaffolds at ratios of 4:2, 4:3, and 4:4 were prepared by mixing SF in a crosslinked gelatin solution in 1:1 ratio. The SF solution was heated at 50–60 °C; then, a glutaraldehyde crosslinked gelatin solution was prepared by adding glutaraldehyde solution (0.25 wt %) into the gelatin solution at a ratio of 1:32 (*v*/*v*). Human hepatocytes and endothelial cells were co-cultured on bioinspired hepatic lobule composite SFC scaffolds using SF-collagen I (SFC) materials via the lyophilization procedure. The SFC scaffold exhibited radially oriented lamellar sheets that mimics the native liver matrix followed by enhancing physiochemical properties and improved biocompatibility. In contrast to the monoculture, the dynamically co-cultured human hepatocytes and endothelial cells in the scaffolds demonstrated a biomimetic radially structured interphase organization pattern and well-maintained functioning that resulted in better polarity and bile capillary development that could be observed in both in vitro and in vivo [135]. Kundu et al. utilized *Antheraea assamensis* non-mulberry silk gland protein fibroin for its biocompatibility, biodegradability, ease of synthesis, and RGD sequence. Due to its ideal properties, silk was used to make cryogels at −20 °C to −80 °C. Cryogels were prepared using 5% SF; it was poured into a mold and kept at −20 °C and −80 °C for 6 h. The frozen SF was immersed in ethanol to remove solvent and was kept at −20 °C overnight. The gelled cryogel was lyophilized to get porous and stable cryogels. Cryogelation may incorporate polystyrene microparticles that are capable of absorbing a variety of liver toxins such as bilirubin, bile acid, and aromatic amino acids, among other liver poisons. Solubilizing fibroin with anionic sodium dodecyl sulphate makes gelation easier by increasing ß-sheet formation. The addition of ethanol made the product porous. Human hepatocytes (HepG2) in cryogels at −20 °C show long-term stable development compared to that observed at −80 °C [136]. Primary human hepatocytes (PHHs) are restricted in their ability to form an in vitro liver model due to dedifferentiation and the loss of liver function, such as cytochrome p450 activity and carrier facilities. They have still been employed in 2D cultures by enhancing culturing techniques such as the spheroid system, co-culture models of liver cell types, and sandwich culture; nevertheless, there are still an extremely large number of problems that need to be solved. Therefore, in vitro tests using immortalized HepaRG cells have shown reliable liver function with cytochrome p450 enzymes in comparison to PHH that has been grown. In order to facilitate the expansion of HepaRG cells, 3% silk was used into the construction of the liver model. The findings showed that the ECM components of silk scaffolds, such as cell binding peptides RGD and type I collagen, substantially influenced CYP3A4 expression and albumin secretion. This resulted in enhanced metabolic activity and albumin secretion [137]. Electrospinning was used to create a biomimetic ECM for hepatic tissue engineering. This biomimetic artificial ECM mimics the architectural traits and biochemical components of natural ECM. Electrospinning was chosen since it can produce nanometer-scale fibers with a high surface to volume ratio and mimics physical properties of natural ECM. Hepatocytes contain asialoglycoprotein (ASGP) receptors that bind to galactose, N-acetyl galactosamine, or glucose residues. Chitosan is biocompatible, biodegradable, antimicrobial, and similar to natural ECM glycans. As a result, galactosylated chitosan (GalCS) and regenerated silk fibroin (RSF) were chosen as the materials of choice and poly(ethylene oxide) (PEO) was utilized to obtain smooth uniform size fibers [138]. Hereby, we have presented some examples of the progression from straightforward in vitro models to more complex 3D constructed liver systems, along with a discussion of the possible implications of these models in drug development and regenerative medicine.

### 6.3. Bioinspiration of Ocular Tissue Engineering

Medical technology innovations are improving the prevention, diagnosis, and treatment of vision loss and hereditary blindness as well as blindness caused by trauma, cataract, glaucoma, diabetic eye disease, or age-related macular degeneration. Ocular disease is responsible for millions of cases of blindness worldwide. Silk has lately gained attention as a bioinspired material of interest for tissue engineering applications requiring regeneration, repair, or replacement of different ocular components [139]. The benefit of silk’s transparency prompted Liu et al. to use silk fibroin in place of the human amniotic membrane, which may avoid these potential challenges of transparency, mechanical stability, and disease transmission [140]. Transparency depends on a number of factors, one of which is the orientation and arrangement of individual cells [141,142]. It has been established that the topographic characteristics on the silk surface, notably the groove pattern, may assist to orient human corneal stromal stem cells and human corneal fibroblasts throughout the process of generating a corneal-like stromal construct. Additionally, it was evident that the incorporation of RGD characteristics into the silk facilitates the expansion and multiplication of corneal stromal cells [139,143,144]. In another research, patients with a deficit in their limbal stem cells exhibited increased neovascularization as well as diminished corneal clarity. A silk film blended with polyethylene glycol (PEG) followed by PEG removal resulted in silk fibroin with enhanced surface roughness and tensile strength, producing a silk fibroin coating that was robust enough to withstand surgical manipulation [145]. Studies on a rabbit model of limbal stem cell deficiency revealed that the limbal epithelial stem cell/silk fibroin grafts inhibited the formation of new blood vessels and restored the corneal epithelium. This provides evidence that cultured limbal epithelial stem cells can differentiate into normal corneal epithelium to replace damaged or diseased epithelium [146]. The Descemet’s membrane is a thin layer of proteoglycans and collagen. The Descemet’s membrane (DM) contributes to the preservation of corneal endothelial phenotype and function. The basement membrane of the corneal endothelium anchors the scaffolds morphogenesis. Any severe damage can cause dystrophies, bullous keratopathy, keratoconus, primary congenital glaucoma, and Fuchs’ dystrophy, all of which end in transparency loss. Silk fibroin has been proven in studies to be an efficient artificial Descemet membrane for the ex vivo cultivation of corneal endothelial cells in preparation for a silk fibroin endothelial graft, resulting in ocular endothelial cell regeneration and function restoration [147,148,149]. The in-depth examination of the internal architecture and distribution of collagen in the stroma inspired the researchers to manufacture silk film-based microarchitecture in order to create a corneal analogue. In addition, silk’s optimal levels of transparency and mechanical integrity, as well as its biocompatibility and controlled degrading qualities, indicate that it may be used as a corneal biomaterial. In a biomimetic technique, silk protein sheets were used to replicate the architecture of corneal stromal tissue. To stimulate the proportions of corneal collagen lamellae, several silk films with the same thickness as corneal collagen were stacked [150,151]. For the trans-lamellar delivery of nutrients and to promote cell-to-cell interaction, each film was perforated with micrometer-sized holes. These films produced encouraging results when evaluated for human and rabbit corneal fibroblast proliferation, alignment, and corneal extracellular matrix expression. In addition, these films can support the functioning of corneal cells, lending greater evidence to the existence of these advantages [150]. Tonsomboon and Oyen found that aligned electrospun gelatin-alginate composite nanofibrous scaffolds had a comparable level of corneal mechanical strength [152]. However, studies showed the utilization of a hazardous chemical crosslinker in order to maintain the scaffold in position. In addition, they have not conducted any tests on their scaffold regarding cellular compatibility. Therefore, the team fabricated a silk-gelatin-based nanofibrous scaffold, which depicted nanofibrous structures with the majority of fibers in a partially aligned manner. The diameter of the fibers ranged from 40 to 320 nm, which is comparable to the diameter of a pair of the native collagen fibers found in the cornea. The findings contribute to a promising research approach towards the fabrication of a corneal stromal equivalent [89].

### 6.4. Bioinspiration of Skeletal Muscle Engineering

The skeletal muscle is one of the three major human muscles. Each skeletal muscle has thousands of fibers encased in connective tissue [153]. The skeletal muscle is responsible for movement, locomotion, breathing, and posture/balance of the body. Skeletal muscles have the ability to repair on its own in response to less severe injuries; nevertheless, more severe injuries and myopathies can cause an irreversible loss of muscle mass and function, e.g., myopathy, paralysis, urinary/bowel incontinence, ataxia, weakness, tremors, and myasthenia gravis. Cell therapies, despite their potential, have not shown a consistent benefit, most likely because the cells that are supplied to the construct do not survive very well. The presentation of chemical and physical signals to muscle cells by biomaterials that are designed to imitate the natural cascade of regeneration can lead to improved muscle regeneration [154]. Researchers have explored the ability of mulberry and non-mulberry silk fibroins to enable myoblast proliferation, differentiation, and myotube creation. The scaffolds fabricated using mulberry silk (*B. mori*) fibroin had an elasticity that was closer to that of muscles (around 12–16 kPa), and as a result, they were best suited to support the formation and alignment of myotubes. Human skeletal muscle myoblasts (HSMMs) were able to adhere, expand, and deposit substantial extracellular matrix (ECM) on each of the silk-based scaffolds. *Bombyx mori* fibroin exhibited long and well-aligned myotubes whereas antheraea mylitta fibroin-based scaffolds demonstrated thicker and shorter myotubes, enabling silk fibroin a suitable material for myotube formation [155]. In order to get a deeper understanding of the processes that lead to muscle injury and degradation, it is essential to develop reliable in vitro models of skeletal muscle. To represent native skeletal muscle tissue, a cell culture model should be three-dimensional, encourage unidirectional myofiber alignment, and be attached at either end of the fiber without relying on a surface for support. Native and transgenic spider silk/silkworm silks were implanted with C2C12 myoblasts to resemble skeletal muscle tissues in vitro. Silks woven around an acrylic chassis produced a unique, three-dimensional cell culture system with suspended muscle fibers that mimic natural skeletal muscle tissue. A genetic investigation showed an increase in skeletal contractile protein gene expression on silkworm silks, especially transgenic silk. The mechanical characteristics and protein secondary structure composition of silk were correlated with myosin gene expression. This increase in contractile protein gene expression implies that proteinaceous, suspended silk substrates may be desirable for in vitro muscle modelling [156]. Scaffolds with 3D porous architectures may also be utilized as carriers to provide an appropriate milieu for transplanted cells for their survival and proper functioning. Natural polymers (alginate, fibrin, and collagen) are widely used in skeletal muscle tissue engineering due to their cell adhesion affinity and intrinsic bioactivity. However, being natural polymers, their degradation is not controlled, making them less ideal for cell protection in the long term. The sericin component of cells also has properties similar to other natural biomaterials. In addition, the fact that sericin does not degrade over a long period of time is an additional advantage that offers the opportunity for an extended time that is adequate for myoblast fusion and bioactivity. To accelerate tissue recovery after severe skeletal muscle damage, Song et al. created a sericin patch releasing miR29-modified myoblasts (SPEED) for fast regeneration and functional repair. MiRNA-29-enriched extracellular vesicles (miR29-EVs) increased myoblast differentiation into myotubes rather than myofibroblasts. For preparing a 13% sericin patch, *w*/*v* sericin was crosslinked with 1% genipin, a naturally occurring crosslinker at a ratio of 3:1 (*v*/*v*). The formed hydrogel was poured onto a 96-well plate at 37 °C and kept at −80 °C overnight, after which, it was lyophilized. The obtained patch after lyophilization was sterilized using 75% ethanol. SPEED improves myoblast integration in a mouse muscle damage model. The muscular milieu improved dramatically 9 days after SPEED implantation, resulting to structural and functional regeneration of TA muscles. Sericin patches improved muscle microenvironment of tibialis anterior (TA) muscles by supporting adhesion and proliferation of myoblasts, promoting myoblast integration into the host (TA), inhibiting CDC20 expression in myocytes, and enhancing myofiber regeneration, thereby suppressing fibrosis and restoring muscle structure and function. Therefore, it was evident that SPEED could be an effective and quick option for treating severe skeletal muscle injuries in mice studies [157].

### 6.5. Bioinspiration of Skin Tissue Engineering

The skin is the outer covering, also known as the integument, of the surface of the body that protects the body from the outside world, and it absorbs sensory stimuli from that outside world. Any trauma or injury may result in chronic or acute wounds. Chronic wounds result in high amounts of proteases and less cytokines and growth factors than acute wounds. Burn injuries cause extensive capillary damage. In diabetic foot ulcers, wound healing cannot advance to the remodeling phase due to additional causes. Concerning the issues stated, it is crucial to identify methods for rapid wound closure with intrinsic skin appendages. Bioinspired replacements and skin grafts that preserve moisture in the wound bed will ensure good re-epithelialization. An ideal skin transplant must be able to imitate the native extracellular matrix (ECM), especially its biological and physiological features. An ideal scaffold’s microstructure would comprise precise chemical components, an ECM-imitating architecture, an optimal porosity, a high surface to volume ratio, interconnected pores with controlled pore shape and size, and adequate mechanical characteristics. Wang et al. reported a potential instance of bioinspiration-based silk microarchitecture. They fabricated nanostructured elastomers that exhibit highly nonlinear mechanical properties. A new controlled radical polymerization method, based on electron transfer atom transfer radical polymerization (ARGET ATRP), is used to synthesize brush polymers with a stiff cellulose backbone and elastic polyisoprene brushes. Furthermore, to form crosslinked brush polymers, ARGET ATRC (activator regenerated by electron transfer atom transfer radical coupling) was used to crosslink the brush polymers at the chain ends of polyisoprene brushes (CBPs). Similar to the morphologies observed in other brush polymer systems, the amphiphilic CBPs form stiff cellulose nanospheres and an elastic polyisoprene matrix. Following the conversion of cellulose nanospheres into cellulose nanofibers by cyclic tensile deformation, these are dispersed in a polyisoprene matrix, replicating the microstructure of human skin [158]. A novel bio-inspired silk fibroin-based microneedle was fabricated in PDMS mold (template) for safe, fast, and easy insertion into the skin tissue. This design strategy offers minimal insertion pain and tissue damage by reducing the insertion force and increasing retraction force. The ability of the silk-made microneedle was verified on a paraffin membrane that acted as a substitute for native skin tissue [159]. Zhang et al. studied the impact of silk fibroin films on full-thickness skin defects in vivo. Rabbit and porcine models were employed for short- and long-term evaluations of wound size, histological investigations, and wound closure at different time periods. The scientists showed that fibroin devices reduced the time of wound healing in rabbits and pigs. A randomized single-blind clinical research on 71 patients revealed silk fibroin films reduced wound healing time and adverse effects compared to conventional patches [160]. A functional living skin was made via digital light processing-based 3D printing, biomimetic hydrogel bioink (GelMA-hyaluronic acid-butanamide-lithium phenyl-2,4,6-trimethylbenzoylphosphinate), and functional cells. This functional living skin supported long-term cell survival, migration, and proliferation. The cantilever beam construction accelerated wound healing by promoting oxygen/nutrient perfusion, metabolic waste elimination, and tissue regeneration. In vivo studies indicated that functional living skin displayed improved skin rejuvenation with follicles in large and small animal models [161]. Electrospinning was used to make an anisotropic membrane. Silk fibroin (SF) and polycaprolactone (PCL) were used to make the top layer thick and waterproof. The dermis-like bottom layer was made of SF and hyaluronic acid with thymol (THY). The generated electrospun anisotropic membrane has the porosity, wettability, and mechanical qualities needed for healing. In vitro results showed that human fibroblast may attach and proliferate across membranes, proving their biocompatibility. THY in the bottom membrane layer increased its antioxidant and antibacterial capabilities. According to the findings, the membrane is an effective choice for wound dressings.

### 6.6. Bioinspiration of Silk in the Area of Healthcare Applications

Over the course of the past several decades, significant developments in biomaterial research have led to the development of game-changing technologies in the field of medical care. This has given rise to the search for low-cost healthcare solutions, with a particular emphasis on renewable biomaterials that have a wide range of uses and are manufactured using environmentally friendly processes. Silk has attracted a lot of attention as a biopolymer, and this may be primarily attributable to the substantial characteristics such as high mechanical strength, biocompatibility, biodegradability, transparency, inexpensiveness, and highly available sources. Silk has been used as a bioinspired material for several drug delivery applications, such as, in a human orthotopic breast cancer model, it was demonstrated that *B. mori* silk films were successful in delivering doxorubicin when the drug was given intratumorally. The influence of doxorubicin-loaded silk films on initial tumor development and metastasis was evaluated in a humanized orthotopic breast cancer model in mice (adenocarcinoma). Both soluble and stabilized silk films loaded with doxorubicin demonstrated a better primary tumor response than the same intravenous dosage. Stabilized silk films coated with doxorubicin decreased primary tumor growth and metastatic dissemination, with no local or systemic toxicity [162]. It was also revealed that β-sheet/silk crystallinity could control the drug release rate. Silk fibroin nanoparticles encapsulated with the medication paclitaxel were shown to be non-cytotoxic and promising drug carriers in a model of gastric cancer by Wu et al. [163]. Patil and colleagues proposed flexible and soft, non-resorbable silk electrode arrays for a neural tissue interface, specifically peripheral nerve, and the neural cortex. The fabricated silk bionic comprised two components i.e., SILK-SEAL and QUICK-SILK. Silk is an FDA-approved biopolymer and was utilized to fabricate a soft and water-resistant silk sandwich sensor known as SILK-SEAL. The QUICK-SILK concept used a silk substrate layer and a specially formulated elastomeric glue to bind silk electrode arrays to the area of interest. This allowed stable electrical recordings from the sciatic nerve and brain. QUICK-SILK can monitor and/or modulate in vivo electrical activity at even but curved surfaces without damaging target tissues/organs [164]. Zhuang et al. created a film made of RSF/PEDOT–OH that has excellent electroconductive characteristics as well as transparency. Due to the film’s high level of transparency, it is possible to observe living cells in real time. In addition, in comparison to the unprotected RSF surface, the conductive polymer layer offers rat brain cell line (PC12 cells) an additional conducive environment for attaching to the surface, proliferating, and differentiating into other cell types. Therefore, the conductive transparent biofilm is the perfect candidate for use in nerve tissue engineering as an electrode for the activation of neural networks. Liu et al. demonstrated wearable epidermal sensors that are crucial for the future of customized healthcare. Adhesion between the flexible sensor and skin is crucial for precise, stable readings. We created a microstructured, silk fibroin-based glue for wearable electronics, named as microstructured fibroin adhesive (MSFA). MSFA has a dependable and stable bonding force on skin surfaces, even in humid or moist situations, and may be peeled off without discomfort. MFSA exhibits strong adhesion and its reusability makes it a seamless adhesive for measuring the pulse, body temperature, and chemical sensors. In current scenario of customized healthcare, the MFSA has a significant amount of potential to be utilized in the role of a functional adhesive for a variety of epidermal electronic sensors [165].

## 7. Conclusions and Future Scope

The current study was primarily focused on the many different sources of silk fibroin as well as the pertinent features that have been naturally bestowed onto it. In addition, new advances, progress, and prominent modalities of the healthcare business that entail the use of silk fibroin and sericin have been discussed in the present review. Bioinspired indirect fabrication methodologies provide silk, exceptional processability and improvement characteristics, meeting biological demands. This review addressed conventional silk building components at several levels, concentrating on unique extraction methods. Related possibilities provided by bioinspired silk fibroin biomaterials may shift future polymer processing approaches toward environment conduciveness and durability, thereby inspiring the creation of high-performance deliverables that may have a transformative impact on academia and industry simultaneously.

## Data Availability

Data sharing not applicable since no new data generated.
